# Differences of the condyle relocation range between skeletal class II and skeletal class III patients: a preliminary study

**DOI:** 10.1186/s12903-026-08308-4

**Published:** 2026-04-17

**Authors:** Xinyu Cui, Hongyi Tang, Huazhi Li, Fu Zheng, Youchao Chen, Tong Wu, Cuiying Li, Jiuhui Jiang

**Affiliations:** 1 Department of Orthodontics, Peking University School and Hospital of Stomatology, No. 22 Zhongguancun Avenue South, Haidian District, 100081 Beijing, China; 2 Central Laboratory, Peking University School and Hospital of Stomatology, No. 22 Zhongguancun Avenue South, Haidian District, 100081 Beijing, China

**Keywords:** Condyle relocation range (CRR), Most comfortable forward relocation (MCFR), Physiologically comfortable mandible position, Temporomandibular joint (TMJ)

## Abstract

**Introduction:**

The present study aimed to preliminarily compare the differences of the condyle relocation range (CRR) between skeletal class II and skeletal class III patients.

**Methods:**

Twenty skeletal class II and twenty skeletal class III young adult patients underwent wide-field cone beam computed tomography (CBCT) scans of the maximum intercuspation position (MIP) and most comfortable forward relocation (MCFR). We superimposed the CBCT images in two positions according to the bilateral zygomatic bone surface structure and then measured the dental and skeletal CRRs in three-dimensional space.

**Results:**

The median (interquartile range) dental and skeletal CRRs of skeletal class II malocclusion patients were 8.71 (3.55) mm and 8.47 (4.19) mm, respectively. The median (interquartile range) dental and skeletal CRRs for skeletal class III malocclusion patients were 1.37 (3.43) mm and 1.04 (1.44) mm, respectively. Both the dental and skeletal CRRs of skeletal class II patients were significantly greater than those of skeletal class III patients (*p* < 0.01).

**Conclusions:**

The CRRs of the different skeletal types exhibit obvious differences. Therefore, the range of physiological mandible positions in patients should be given attention during orthodontic diagnosis and treatment.

## Introduction

The temporomandibular joint (TMJ) is one of the movable joints of the human body and the only movable joint in the maxillofacial region. General dentists and specialists are very familiar with the static anatomy of the TMJ. However, there are many different views on its functional anatomy. The prevailing view is that the TMJ is similar to the hip joint, and the relationship between the condyle and the glenoid fossa is similar to that of a delicate ball articulating in a socket. Some people believe that the optimal condyle‒fossa relationship is concentric relationship [1–3], and some orthodontists prefer to deprogram the mandible and reconstruct the maximum intercuspation position (MIP) in accordance with the centric relation position(CRP) [4, 5]. However, others believe that the relationship between the condyle and the fossa is dynamic, for instance, the “long concentric” phenomenon in prosthodontics [6] and “the ball on the hill”, a new perspective on TMJ functional anatomy proposed by the orthodontist Charles S Greene [7, 8].

In daily practice, we may find that skeletal class II patients can occlude in several different positions in the sagittal dimension without discomfort, while the mandibles of skeletal class III patients seem immovable in the sagittal dimension. To our knowledge, previous studies have not confirmed this phenomenon, and very few studies have shown similar effects. Turasi reported that the centric occlusion(CO)-maximum intercuspation (MI) discrepancy in patients with an increased overjet (OJ>6 mm) was greater than that in people with normal occlusion [9]. Rainer and Ueki demonstrated that the free mandibular movement of skeletal class II patients and skeletal class III patients is different from that of skeletal class I patients [10]. Besides, correlation between maximal jaw opening and craniofacial morphology was also identified [11]. The above studies suggested that the condyles of different sagittal skeletal types may exhibit different ranges of movement related to glenoid fossae.

We speculated that the range of physiological condyle positions may also vary among different sagittal skeletal types. Therefore, a new concept is developed, the condyle relocation range (CRR), to describe the physiological movement range of the condyle and mandible in the sagittal dimension. Specifically, the CRR refers to the range in which the mandible moves from the MIP to the most comfortable forward relocation (MCFR) position. The CRR is distinct from the range of mandibular border movement [10, 12, 13] and the centric occlusion (CO) -centric relation (CR) discrepancy [9, 14, 15]. MCFR, the anterior border of the CRR, refers to the position at which patients automatically protrude their mandibles as far as they can, while maintaining interocclusal contact between their anterior or posterior teeth and feeling no pain or stretching of their TMJs. The MCFRs of skeletal class II patients could be edge-to-edge positions or even more forward to the anterior crossbite positions. Specifically, the displacement of the condyle is called the skeletal CRR, and that of the lower central incisors is called the dental CRR.

Based on our clinical observation and the relevant studies, we come up a hypothesis: There may be a great difference of the CRR between skeletal class II and skeletal class III patients, which may have a certain prospect of clinical application. Therefore, the difference of the CRR between skeletal class II and skeletal class III patients was investigated quantitatively in the present study.

## Materials and methods

Based on the mandibular border movement (MBM) data from a previous study [10], a power calculation indicated that 14 patients were needed in each group to achieve a confidence level of 0.95 and a probability of 0.8, for a total of 28 patients all (t = (x1 - x2)/[s^2 * (1/n1 + 1/n2)]^0.5, set n1 = n2). x1 and x2 represent the average of the two samples; s^2 represents the weighted average of the variances of the two samples (combined variances); and n1 and n2 represent the sample sizes of the two samples. Considering the difference between the MBM and the newly defined CRR, we expanded the sample size to 40 patients, 80 condyles, and each group contained 20 patients, 40 condyles.

We recruited 20 skeletal class II and 20 skeletal class III young adult patients with treatment need of dentofacial deformity who visited the Department of Orthodontics or Orthognathic Surgery, Peking University Hospital of Stomatology, from March 1 to December 1, 2021. The inclusion criteria were as follows: (1) skeletal class II diagnosed by an ANB angle > 5° or skeletal class III diagnosed by an ANB angle < 0°; and (2) no symptoms of temporomandibular joint disorder [16] (including pain, discomfort, restricted mouth opening, or visible bone changes on images of the TMJ area and oral parafunctions such as bruxism). The exclusion criteria were as follows: (1) orthodontic treatment history; (2) anterior open bite; (3) unilateral posterior crossbite and scissors bite; (4) severe facial deviation; and (5) craniofacial development syndrome and cleft lip and palate. The study was approved by the biomedical ethics committee of Peking University Hospital of Stomatology and the ethics approval document number was (PKUSSIRB-202162020), and was conducted in accordance with the Helsinki Declaration of 1975, as revised in 2013. Participant consent was obtained from all the involved patients. The process of the experiment is described below, and all the subjects completed the experiment. Clinical trial number: not applicable.

### Registration of MCFR

Patients were instructed to protrude their mandibles forward voluntarily as far as they could until the anterior teeth reached the edge-to-edge position and then even further forward to the anterior crossbite position, while maintaining interocclusal contact and feeling no pain or stretching in the TMJ region. After the patients had practiced three times, the MCFR was confirmed, and the researcher injected some silicone rubber bite recorder (O-Bite, Dmg, Hamburg, Germany) on the occlusal surface of their bilateral posterior teeth to register their MCFR.

### Wide-field CBCT ( Newtom Ag, Marburg, Germany) acquisition at the MCFR and MIP

**Fig. 1:**
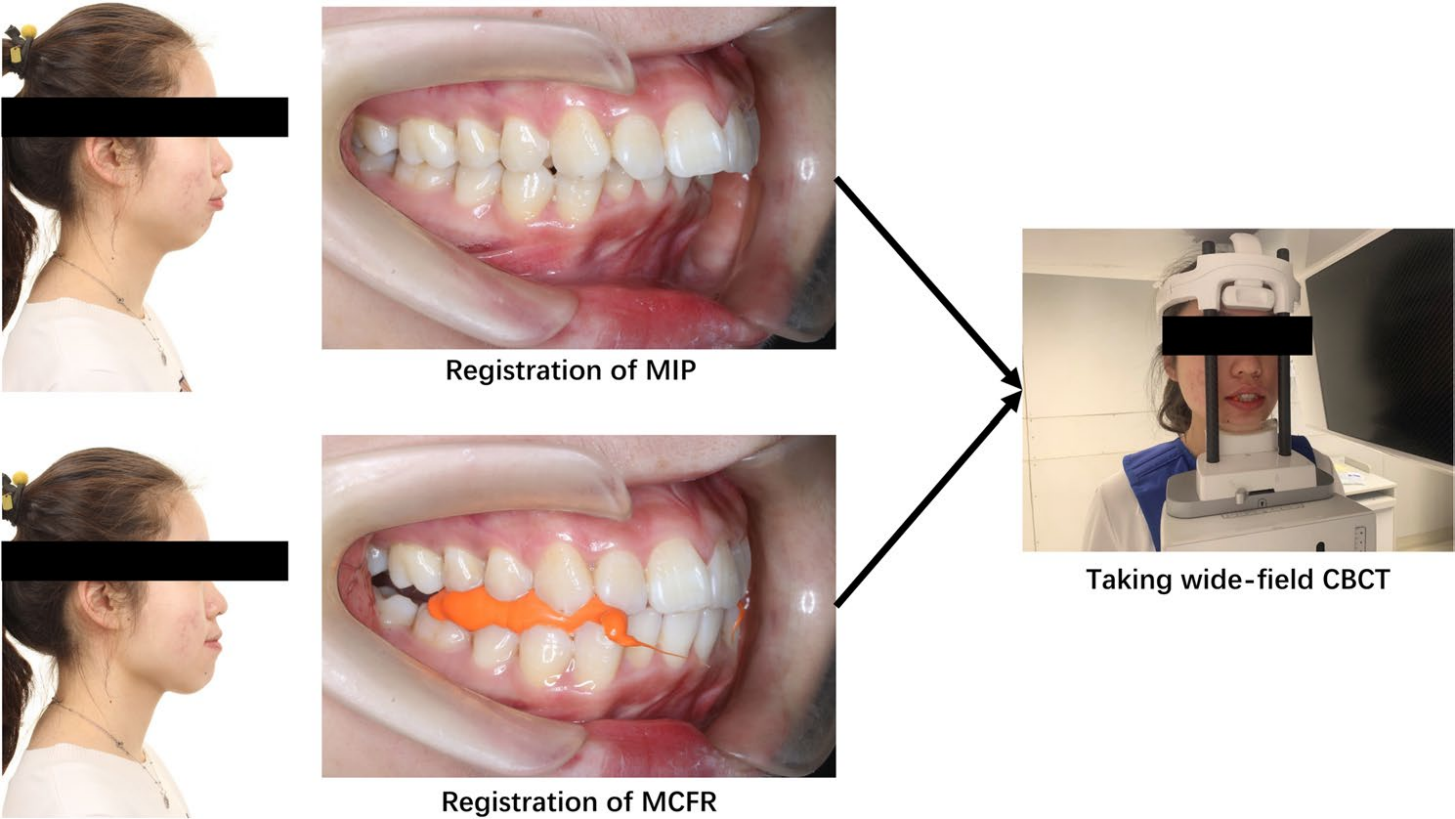
After registration of MIP and MCFR, patients underwent wide-field CBCT

The CBCT parameters were as follows: tube voltage, 110 kV; tube current, 2.03 mA; scan field, 15 × 15 cm; axial slice thickness, 0.3 mm; and exposure time, 3.6 s. First, patients underwent a CBCT scan with MCFR registration for research purposes. Second, they were instructed to remove the intraoral silicone rubber bite recorder and occlude in the most stable and interdigitated position (MIP). Third, an additional CBCT scan was acquired at MIP for radiographic assessment as part of their pre-orthodontic or pre-orthognathic evaluation (Fig. 1).

### Synthesis and analysis of cephalograms

**Fig. 2:**
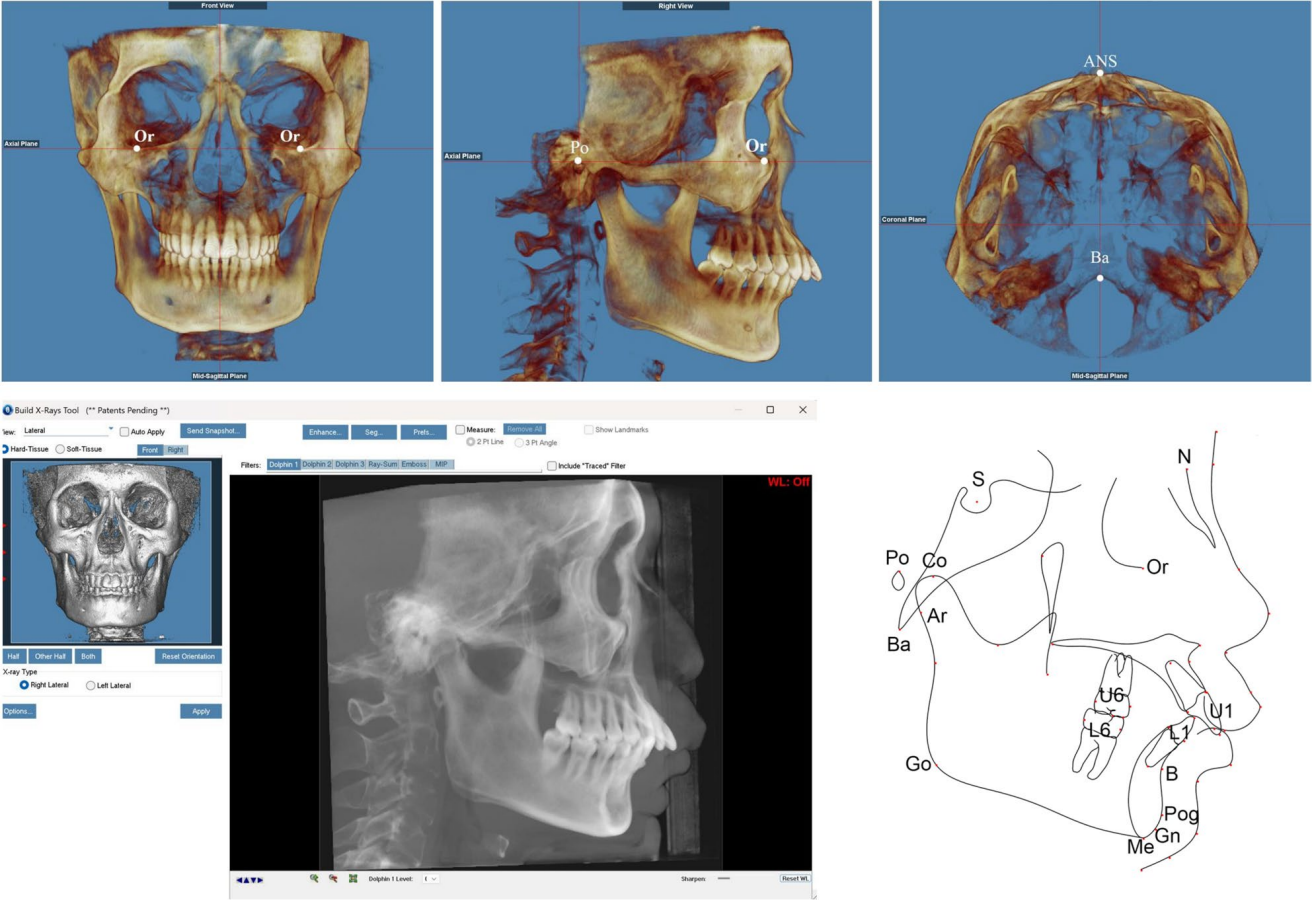
Synthesis and analysis cephalograms using dolphin imaging software

In Dolphin Imaging Software (Dolphin Imaging & Management Solutions, California, America), the 3D images were reoriented, that the bilateral FH plane was parallel to the horizontal plane, and the midsagittal plane passed through the basion point (Ba) and anterior nasal spine (ANS) at the same time. Lateral cephalometric images were obtained by Dolphin software using the parallel projection method with the direction from right to left. Next, we measured the dental and skeletal craniofacial features of the MI and MCFR positions of each patient (Fig. 2 Table 1).

**Table 1:** Descriptions and definitions

Cephalometric parameters	
S	Sella, the midpoint of the cavity of sella turcica
N	Nasion, the anterior point of the intersection between the nasal and frontal bones
A	the innermost point on the contour of the premaxilla between anterior nasal spine and the incisor tooth
B	the innermost point on the contour of the mandible between the incisor tooth and the bony chin
Po	Porion, the midpoint of the upper contour of the external auditory canal (anatomic porion)
Or	Orbitale, the lowest point on the inferior margin of the orbit
Pog	Pogonion, the most anterior point on the contour of the chin
Go	Gonion, the midpoint connecting the ramus and body of mandible
Me	Menton, the most inferior point on the mandibular symphysis—that is, the bottom of the chin
SN	anterior basal plane, the plane connecting S and N
FH	Frankfort horizontal plane, the plane connecting Or and Po
MP	Mandibular plane, the plane connecting point Go and point Me
SNA (°)	The angle between point S, point N and point A
SNB (°)	The angle between point S, point N and point B
ANB (°)	The angle between point A, point N and point B
Facial Angle (FH-NPog) (°)	the angle between FH plane and N-Pog line
Convexity (NA-APog) (°)	the angle between N-A line and A-pog line
U1 - NA (mm)	Perpendicular distance from the U1 to N-A line
U1 - NA (°)	the angle between the long axis of the upper incisor and N-A line
L1 - NB (mm)	Perpendicular distance from the L1 to N-B line
L1 - NB (°)	the angle between the long axis of the lower incisor and N-B line
Interincisal Angle (U1-L1)(°)	the angle between the long axis of the upper and that of lower incisor
SN - MP (°)	The angle between MP plane and SN plane
FMA (MP-FH) (°)	The angle between MP plane and FH plane
CRR parameters	
Dental CRR	the distance between Li in MIP and MCFR
Dental CRR-x	the horizontal component of Dental CRR
Dental CRR-y	the vertical component of Dental CRR
Dental CRR-z	the anteroposterior component of Dental CRR
Skeletal CRR-R	the distance between CC in MIP and MCFR-right condyle
Skeletal CRR-R-x	the horizontal component of Skeletal CRR-right condyle
Skeletal CRR-R-y	the vertical component of Skeletal CRR-right condyle
Skeletal CRR-R-z	the anteroposterior component of Skeletal CRR-right condyle
Skeletal CRR-L	the distance between CC in MIP and MCFR-left condyle
Skeletal CRR-L-x	the horizontal component of Skeletal CRR-left condyle
Skeletal CRR-L-y	the vertical component of Skeletal CRR-left condyle
Skeletal CRR-L-z	the anteroposterior component of Skeletal CRR-left condyle

### Measurement of the dental and skeletal CRRs

#### Reconstruction of a 3D model of the full craniofacial bone

**Fig. 3:**
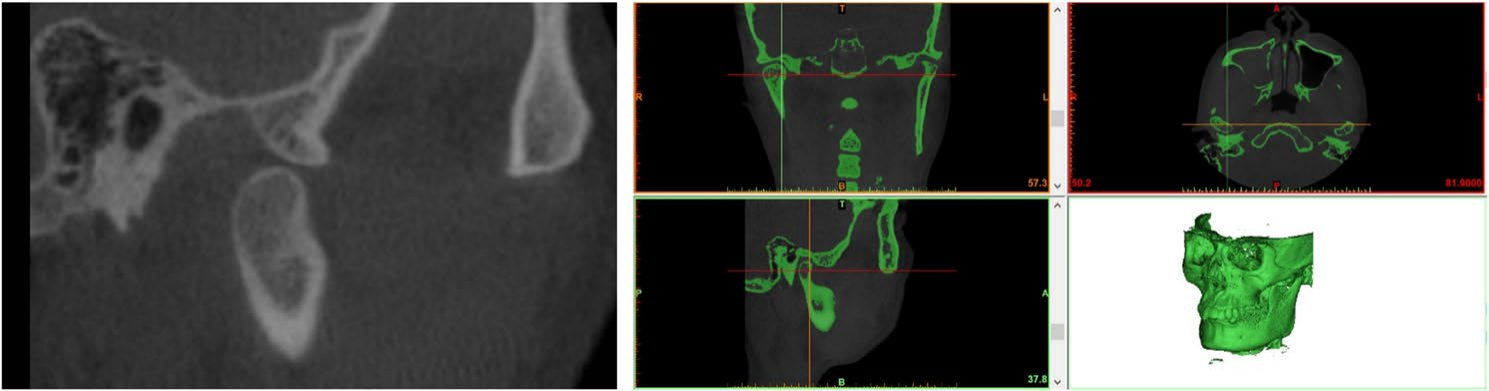
3D reconstruction of CBCT using mimics software

In Mimics Medical 20.0 Software (Materialise, Brussels, Belgium), the full craniofacial bone of MI and MCFR positions was extracted with the gray value of the condyle (approximately 400 HU), reconstructed into 3D models and subsequently saved in STL format (Fig. 3).

#### Superimposition of 3D models

**Fig. 4:**
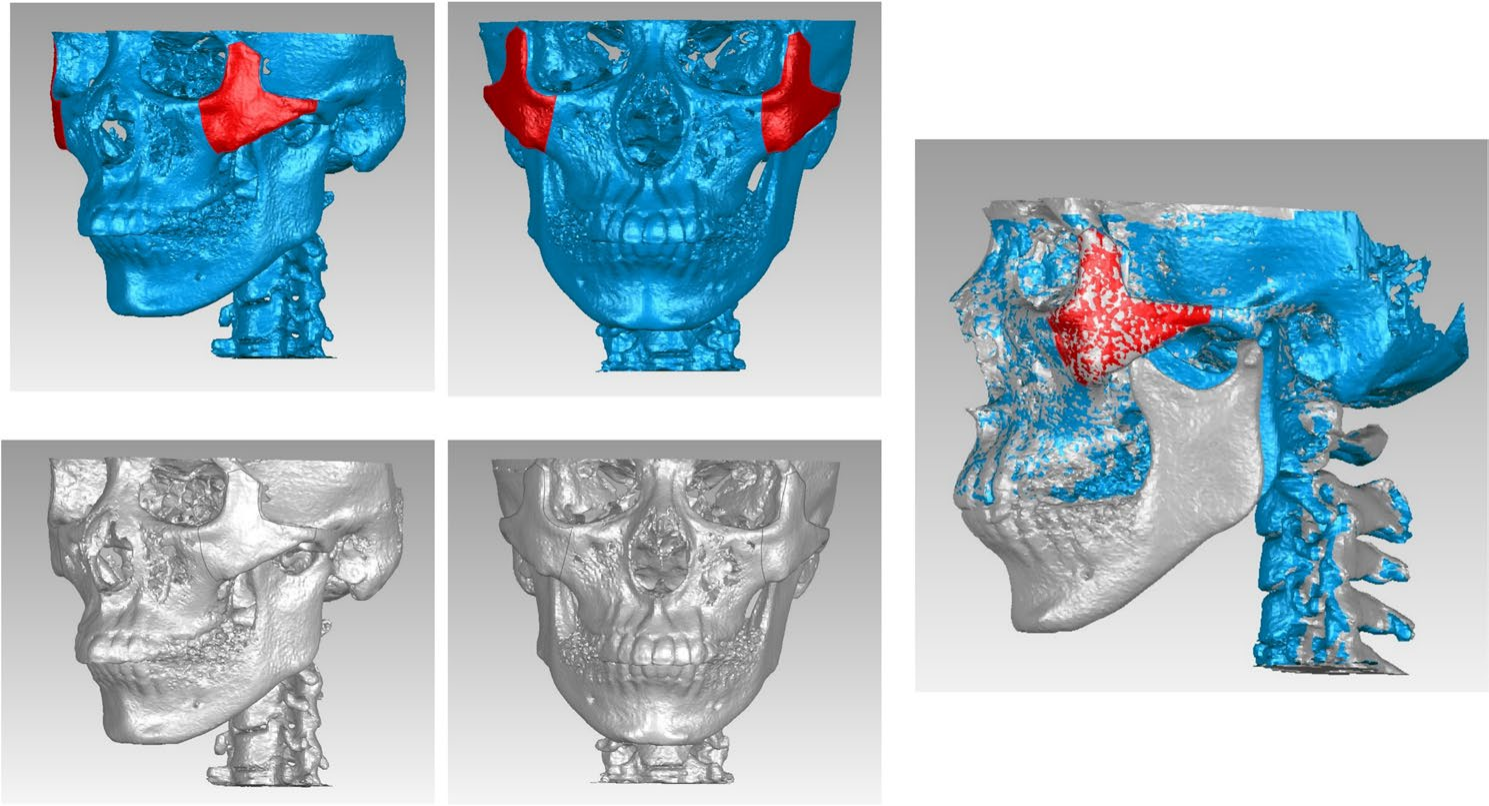
Superimposition of reconstructed 3D models by bilateral zygomatic bones

**Fig. 5:**
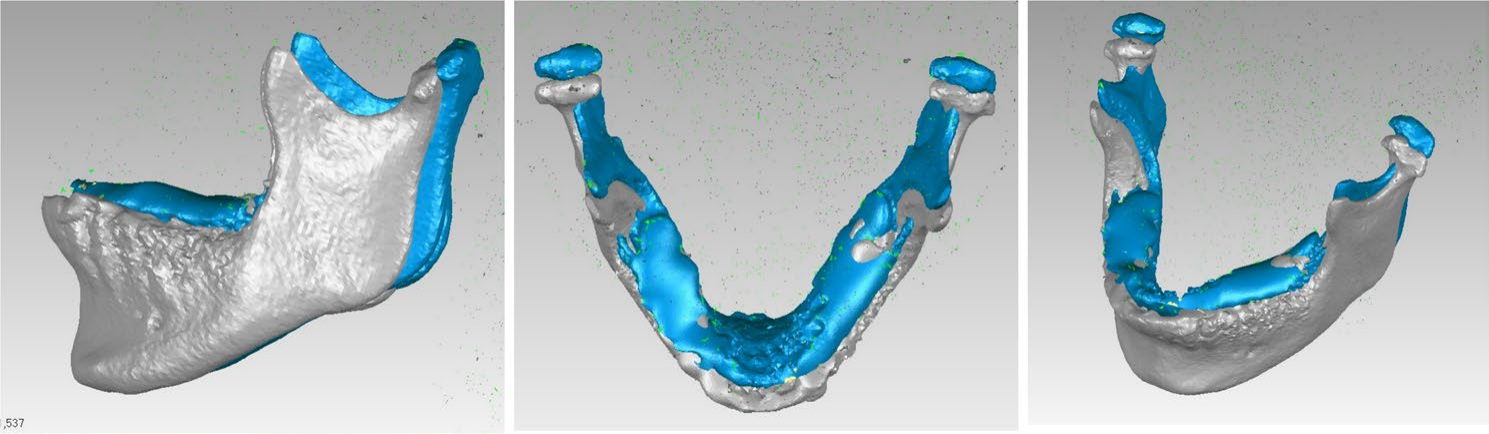
Reconstructed mandibles in MIP( colored blue) and MCFR( colored grey)

After importing the reconstructed full craniofacial bone 3D model into Geomagic Studio 2013 Software (Geomagic, North Carolina, America). We sketched the contour of the bilateral zygomatic bones (inferior orbital border, zygomaticomaxillary suture, inferior border of the zygomatic arch, zygomatic temporal suture, superior border of the zygomatic arch); then, we superimposed the full craniofacial bone 3D models of the MCFR and MI positions according to the bilateral zygomatic bone surface (Figs. 4 and 5).

#### Location of mark points (Figs. 6, 7 and 8; Table 1)

**Fig. 6:**
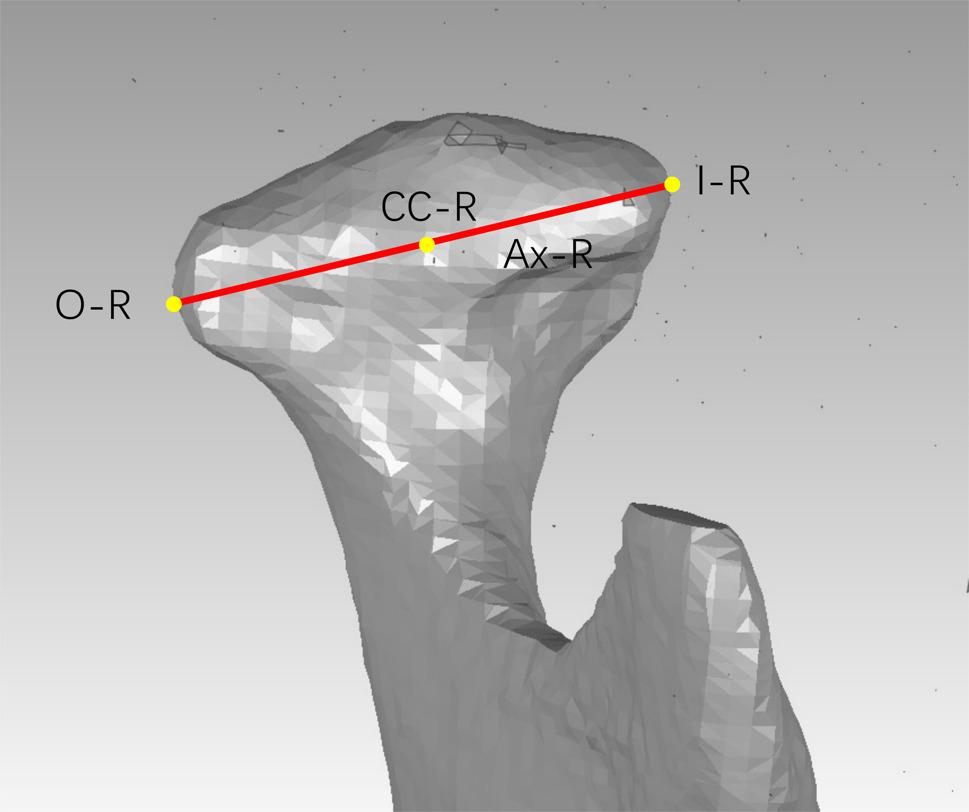
A reconstructed right condyle with mark points. Condylar innermost point-right condyle(I-R); condylar outermost point-right condyle(O-R); axial of condyle-right condyle( Ax-R) ; geometric center of condyle- right condyle(CC-R)

**Fig. 7:**
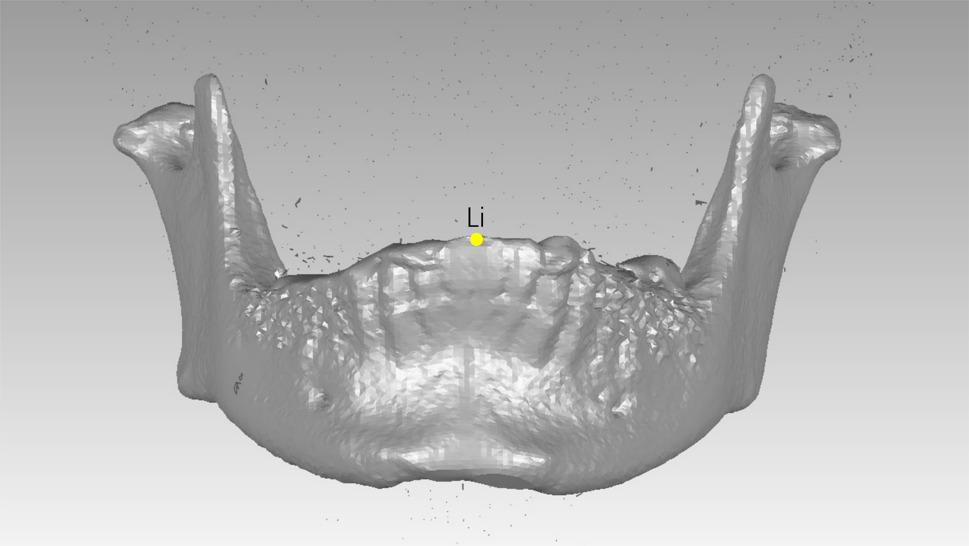
A reconstructed mandible with a mark point lower incisor point (Li)

**Fig. 8:**
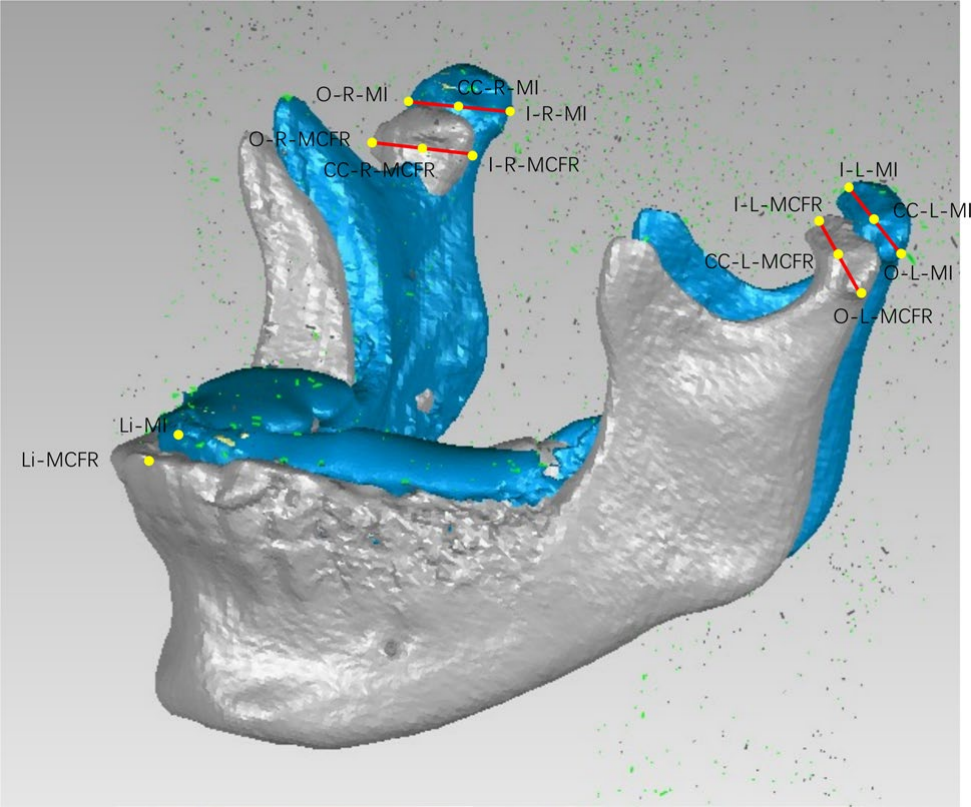
Reconstructed mandibles in MIP and MCFR with mark points. Geometric center of condyle- right condyle in MI (CC-R-MI); condylar outermost point-left condyle in MCFR(O-L-MCFR)etc

- Condylar innermost point (I): the most prominent point on the condylar inner surface
- Condylar outermost point (O): the most prominent point on the condylar outer surface
- Axis of condyle (Ax): a line connecting the condylar innermost point and outermost point
- Geometric center of condyle (CC): the midpoint of the axis of the condyle
- Lower incisor point (Li)

#### Measurement of CRR (Fig. 9)

**Fig. 9:**
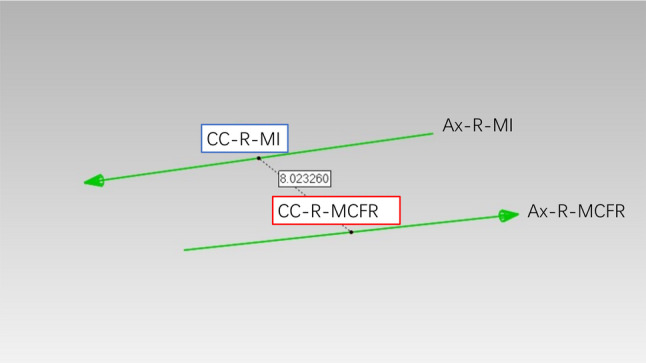
Measurement of skeletal CRR

- Skeletal CCR: the distance between the CC in the MIP and MCFR positions
- Dental CRR: the distance between Li in the MIP and MCFR positions

### Statistical analysis

The statistical analysis was performed using SPSS 23.0 Software (IBM Corp., Armonk, Ny, America). The difference in the sex ratio between groups was compared by the chi-square test. Normality and homogeneity of variance were assessed by the Kolmogorov‒Smirnov test and Levene test, respectively. The age and cephalometric analysis results were normally distributed and were analyzed with an independent t test, while the CRR results were nonnormally distributed and were analyzed with the Wilcoxon rank sum test.

## Results

### Intra- and inter-observer reliability

To test the reliability of the measurements, all the samples were remeasured two weeks after the initial measurement by the same investigator (X.Y.C) and another investigator (T.W). The intraclass correlation coefficient (0.991–0.998) and interclass correlation coefficient (0.964–0.991) showed excellent intra- and inter-observer reliability.

### Baseline demographic characteristics (Table 2)

The average age of skeletal class II patients was 22.04 ± 3.80, and that of skeletal class III patients was 22.13 ± 3.89. There was no significant difference between the two skeletal types (*p* = 0.84). The ratio of females to males in skeletal class II and skeletal class III was 45% and 60% respectively, with no significant difference between the two groups (*p* = 0.34).

**Table 2:** Baseline demographic characteristics

	Skeletal Class II	Skeletal Class III	P value（P<0.05*）
age	22.04±3.80	22.13±3.89	0.84
sex(%)			
women	9(45)	12(60)	0.34
men	11(55)	8(40)	

### Craniofacial characteristic features of skeletal class II and skeletal class III patients in the MI and MCFR positions (Tables 3 and 4)

For skeletal class II patients, in anteroposterior dimension, SNB(°), facial angle, L1-NB (mm), and L1-NB (°) values were greater in the MCFR position than in the MI position, while the ANB and angle of convexity were smaller in the MCFR position than in the MI position (*p* < 0.001). In vertical dimension, SN-MP of skeletal class II patients in the MCFR position was smaller than that in the MI position (*p* = 0.035).

**Table 3:** Comparison of Craniofacial characteristic features of skeletal class II in MI and MCFR position

Cephalometric parameters	MI	MCFR	P value
SNA (°)	82.66±2.84	82.99±2.75	0.794
SNB (°)	74.93±3.06	79.49±3.65	0.00*
ANB (°)	7.72±2.33	3.52±2.56	0.00*
Facial Angle (FH-NPog) (°)	85.18±4.02	88.83±4.98	0.00*
Convexity (NA-APog) (°)	15.33±6.35	6.87±6.52	0.00*
U1 - NA (mm)	6.45±2.93	9.73±9.23	0.255
U1 - NA (°)	27.02±8.31	25.36±9.365	0.811
L1 - NB (mm)	9.17±2.21	14.98±11.53	0.00*
L1 - NB (°)	34.03±5.80	49.35±28.94	0.00*
Interincisal Angle (U1-L1)(°)	111.24±8.20	110.57±8.65	0.691
SN - MP (°)	37.97±8.57	35.15±7.68	0.035*
FMA (MP-FH) (°)	26.14±8.34	36.90±29.11	0.248

Values are presented as mean ± standard deviation

*P<0.05

**Table 4:** Comparison of Craniofacial characteristic features of skeletal class III in MI and MCFR position

Cephalometric parameters	MI	MCFR	P value
SNA (°)	80.44±5.22	79.12±4.45	0.089
SNB (°)	85.08±4.85	84.24±5.33	0.257
ANB (°)	-4.64±2.51	-5.10±2.60	0.337
Facial Angle (FH-NPo) (°)	95.58±5.08	95.88±5.06	0.492
Convexity (NA-APo) (°)	-10.76±6.00	-11.57±6.54	0.352
U1 - NA (mm)	8.62±2.22	8.57±2.12	0.744
U1 - NA (°)	34.57±5.57	34.13±5.61	0.42
L1 - NB (mm)	4.37±2.53	4.62±2.47	0.111
L1 - NB (°)	19.70±5.67	20.64±6.55	0.101
Interincisal Angle (U1-L1)(°)	130.36±7.58	130.34±8.33	0.695
SN - MP (°)	33.39±7.65	35.71±9.36	0.007*
FMA (MP-FH) (°)	22.61±8.15	22.90±8.71	0.601

Values are presented as mean ± standard deviation for normal distributions

*P<0.05

For skeletal class III patients, all the anteroposterior measurements between MCFR position and MI position had no significant difference, while the SN-MP of skeletal class III patients in the MCFR position was larger than MI position (*p* = 0.007).

### CRRs of skeletal class II and skeletal class III patients (Table 5)

For skeletal class II patients, the median (interquartile range) dental CRR was 8.71 (3.55) mm, and the median (interquartile range) skeletal CRR was 8.47 (4.19) mm. For skeletal class III patients, the median (interquartile range) dental CRR was 1.37 (3.43) mm, and the median (interquartile range) skeletal CRR was 1.04 (1.44) mm. Both skeletal class II and skeletal class III patients had relatively larger vertical component of the dental and skeletal CRRs than that of anteroposterior and horizontal components.

**Table 5:** Comparison of CRR of skeletal class II and skeletal class III

CRR parameters	Skeletal Class II	Skeletal Class III	P value
Dental CRR	8.71 (3.55)	1.37(3.43)	0.00*
Dental CRR-x	0.91(1.25)	0.53(0.97)	0.239
Dental CRR-y	7.65(3.10)	0.30(1.50	0.00*
Dental CRR-z	4.22(2.36)	0.98(1.04)	0.00*
Skeletal CRR-R	8.04(4.42)	0.92 (1.06)	0.00*
Skeletal CRR-R-x	0.51(0.342)	0.29(0.41)	0.00*
Skeletal CRR-R-y	5.79(3.84)	0.75 (0.77)	0.00*
Skeletal CRR-R-z	4.67(2.18)	0.58 (1.21)	0.00*
Skeletal CRR-L	8.90(3.96)	1.17(1.82)	0.00*
Skeletal CRR-L-x	0.45 (0.63)	0.21(0.37)	0.031
Skeletal CRR-L-y	7.015 (3.44)	0.78 (1.49)	0.00*
Skeletal CRR-L-z	4.33(2.35)	0.82(1.68)	0.00*

Values are presented as median (interquartile range)

*P<0.05

The dental and skeletal CRRs of skeletal class II patients were significantly greater than those of skeletal class III patients (*p* < 0.001).

## Discussion

For the first time, we developed the concept of the CRR to describe the potential range of mandible positions under physiological conditions. The CRR is distinct from the range of MBM. The anterior border of the MBM is the most protrusive position (MPP), the most forward mandibular position that is used for chewing or speaking, which is temporary, rather than interdigitating and persistently maintaining the mandible position. The mandible is protruded to the greatest extent in the MPP. However, the anterior border of the CRR is the MCFR, which has two connotations. The first is comfort, which guarantees that the mandibular position within the CRR has no impact on TMJ health and can be stably maintained; that is, the MCFR is a physiological mandibular position. The second is maintaining occlusal contact, which ensures the anteroposterior range of mandibular relocation under the precondition of a specific vertical dimension.

The present study clearly demonstrated that the CRR of skeletal class II patients was greater than that of class III patients, which is consistent with our clinical observations and confirms our hypothesis. These findings deepen our understanding of functional TMJ anatomy and broaden our understanding of human maxillofacial morphology. Giuntini concluded that the posteriorly displaced glenoid fossa is a characteristic of class II malocclusion with mandibular retrusion [17]. Several studies have verified that more anteriorly positioned condyles are present in skeletal class II patients than in skeletal class I and skeletal class III patients [18–20]. Vitral [21] and Li [22] reported that the condyles on the class I side of class II subdivision patients were located more anteriorly than those on the contralateral side. Therefore, we speculate that the condyles of skeletal class II patients are located more anteriorly to compensate for the retrusive mandibles, which are restrained by the posteriorly located glenoid fossa.

In addition, the small size of the condyles of skeletal class II patients [23–27] may facilitate the ability of the mandibles to freely relocate anteriorly, which may constitute the morphological basis of the large CRR in skeletal class II patients. On the contrary, the large size of the condyles [28] and the relatively narrow fossae of skeletal class III patients [29], may be attributed to their small CRR.

**Fig. 10:**
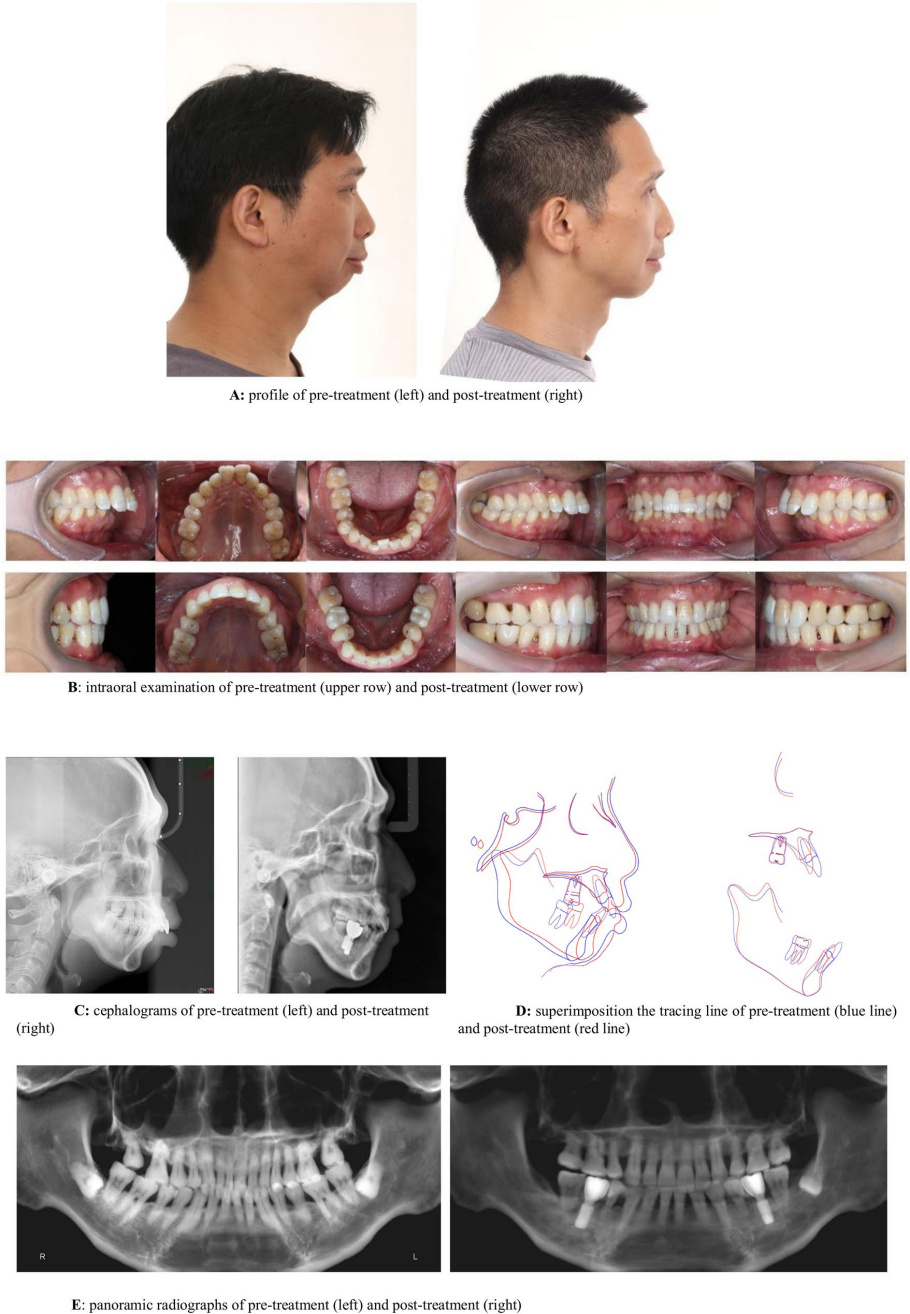
A 41-year-old male with skeletal Class II malocclusion and mandibular retrusion treated with premolar extractions and fixed orthodontics, illustrates the clinical utility of CRR-based forward relocation of the mandible in skeletal Class II malocclusion

Orthodontic treatment involves not only intra-arch tooth movement within the dental alveolar bone but also inter-arch occlusal adjustment, which changes the mandibular position and remodels the TMJ. Fan demonstrated that additional increase of condylar length is the principal skeletal effect of Herbst treatment [30]. Koide reported that the glenoid fossa exhibited remodeling after a four first premolar extraction therapy [31]. Alhammadi investigated a cohort of single jaw extraction cases and reported that the condyles had moved backward [32]. Bayirli and his colleagues demonstrated that the edgewise appliance could improve the pattern of mandibular forward growth displacement through the maintenance of vertical control [33]. In our clinical practice, we have successfully treated numerous skeletal class II patients with retrusive mandibles who refused orthognathic surgery intervention by exploiting their considerably large CRRs. Figures 10 and Table 6 shows a representative case, an adult male patient of retrusive mandible, who refused orthognathic surgery, relocated forward his mandible by orthodontic treatment.

**Table 6:** Cephalometric analysis of pre-treatment and post-treatment

Cephalometric parameters	pre-treatment	post-treatment	normal value	standard deviation
SNA(°)	76.5	77.3	82.0	3.5
SNB(°)	68.4	72.0	80.9	3.4
ANB(°)	8.1	5.3	1.6	1.5
Facial Angle (FH-NPo)	82.9	85.1	89.6	3.0
Convexity (NA-APo)	17.9	10.8	2.5	3.0
U1 - NA (mm)	4.1	1.1	4.3	2.7
U1 - NA(°)	22.3	15.3	22.8	5.7
L1 - NB (mm)	7.8	8.5	4.0	1.8
L1 - NB(°)	31.5	34.1	25.3	6.0
U1 - L1 (°)	118.0	125.3	131.0	7.0
U1 - SN(°)	98.8	92.6	103.1	5.5
MP - SN(°)	49.4	47.3	33.0	6.0
FMA (MP-FH)	31.4	31.5	22.9	4.5
IMPA (L1-MP)	-3.8	-4.8	1.4	3.5
Y-Axis -- Downs (SGn-FH) (°)	65.6	64.4	60	3.4
Pog - NB (mm)	-0.1	0	3	1.7

The CRR is a functional craniofacial characteristic and should be included in pre-orthodontic examinations to facilitate comprehensive assessment of the patients. Exploiting the relatively large CRR, an alternative treatment paradigm for skeletal class II patients was introduced, in which the condyle was relocated forward to compensate for the retrusive mandible rather than causing proclination of the lower incisors. This paradigm is especially applicable for adult patients who refuse orthognathic surgery intervention but have relatively large CRRs. Adolescent skeletal class II patients with large CRRs could also incorporate the CRR into their treatment plan to achieve a better facial profile outcome. Nevertheless, combined orthodontic and surgical treatment is still the best option for skeletal class III patients because of their small CRR.

The use of the condylar position indicator (CPI) is the most common approach for measuring condylar shift. The condyle center is located by an empirically chosen skin surface marker, which is definitely not accurate [9, 14]. Axiography based on ultrasound device have the limitation that recording incisor movement as surrogate [34]. As the condyle and glenoid fossa have irregular geometries, a 3D reconstructed condyle model from CBCT images could comprehensively and accurately reveal the actual stereoscopic structure and unique position of the TMJ [35]. In this study, we developed a condylar displacement measurement method. After reconstructing a 3D model of the full craniofacial bone in the MIP and the MCFR position, we superimposed the two models according to the bizygomatic surfaces and then measured the condylar shift in three-dimensional space.

As a preliminary study, although the present research yielded several noteworthy findings, it is not without limitations. In terms of research methodology, the mandibular protrusion required to achieve MCFR results in stretching of the maxillofacial muscles. As a result, the recorded head position at MCFR may differ from that at MI, which could slightly affect the measurement results of the CRR. Although CBCT is the most direct and accurate method for obtaining the position and morphology of the hard tissues of the condyle at the MCFR, it remains essential to explore safer alternative methods and to optimize experimental protocols. Real-time magnetic resonance imaging (rtMRI) can not only record the condyle-disc-fossa relationship during the whole mandibular movement in real time, but collect more jaw position information data, which may be of great help to our future research [36, 37].

In terms of statistical analysis, although a post-hoc power analysis based on the current CRR data (with 9 subjects per group and a total sample size of 18) confirms that the existing sample size meets statistical requirements, subsequent studies should still expand the sample size to further validate differences in CRRs among various sagittal skeletal types. The multiple cephalometric comparisons were conducted without Bonferroni or FDR correction, which may increase the risk of Type I errors. However, as this is a preliminary exploratory study, its primary objective is to identify potentially meaningful trends and generate hypotheses for future research. To enhance the reliability of the findings, we plan to expand the sample size in subsequent studies. This will provide sufficient statistical power to validate these initial observations using more stringent correction methods.

In terms of in-depth investigation, the relationship of vertical skeletal types and CRRs should be demonstrated as well. As the incidence of temporomandibular joint disease (TMD), is comparatively higher in skeletal class II patients than other sagittal skeletal types [38], the CRRs of skeletal class II patients with TMD is also worth measuring.

## Conclusion

The CRR is a functional craniofacial characteristic that varies among different skeletal types. The CRR in skeletal class II patients is significantly greater than that in skeletal class III patients. CRR measurement should be included in pre-orthodontic examinations, and orthodontists should pay attention to the range of physiological mandible positions.

## Data Availability

All data generated or analyzed during this study are included in this published article.

## Abbreviation

CRR: Condyle relocation range
CBCT: Cone beam computed tomography
MIP: Maximum intercuspation position
MCFR: Most comfortable forward relocation
TMJ: Temporomandibular joint
CRP: Centric relation position
CO: Centric occlusion
MI: Maximum intercuspation
TMD: Temporomandibular disorder
CR: Centric relation
MBM: Mandibular border movement
MPP: Most protrusive position
CPI: Condylar position indicator
rtMRI: Real-time magnetic resonance imaging

## References

[1] Pullinger AG, et al. Relationship of mandibular condylar position to dental occlusion factors in an asymptomatic population. Am J Orthod Dentofac Orthop. 1987;91(3):200–6. 10.1016/0889-5406(87)90447-1 3469906

[2] Rodrigues AF, Fraga MR, Vitral RW. Computed tomography evaluation of the temporomandibular joint in Class I malocclusion patients: condylar symmetry and condyle-fossa relationship. Am J Orthod Dentofac Orthop. 2009;136(2):192–8. 10.1016/j.ajodo.2007.07.032 19651348

[3] Kandasamy S, Greene CS, Obrez A. An evidence-based evaluation of the concept of centric relation in the 21st century. Quintessence Int. 2018;49(9):755–60. 30202837 10.3290/j.qi.a41011

[4] Fornai C, et al. Centric relation: A matter of form and substance. J Oral Rehabil. 2022;49(7):687–90. 35377510 10.1111/joor.13329 PMC9324986

[5] Fornai C, et al. Robert M. Ricketts and Rudolf Slavicek: dentistry by the rules of nature. Angle Orthod. 2023;93(5):497–500. 37503706 10.2319/050423-323.1 PMC10575632

[6] He H, Zhang S, Xu J. Impact of occlusal reconstruction positions on airway dimensions in patients with edentulism and long centric occlusion. BMC Oral Health. 2023;23(1):215. 37060039 10.1186/s12903-023-02931-1 PMC10105404

[7] Greene CS. The Ball on the Hill: A new perspective on TMJ functional anatomy. Orthod Craniofac Res. 2018;21(4):170–4. 30318699 10.1111/ocr.12245

[8] Zonnenberg AJJ, Türp JC, Greene CS. Centric relation critically revisited-What are the clinical implications? J Oral Rehabil. 2021;48(9):1050–5. 34164832 10.1111/joor.13215

[9] Turasi B, Ari-Demirkaya A, Biren S. Comparison of increased overjet cases and controls: normative data for condylar positions. J Oral Rehabil. 2007;34(2):129–35. 17244235 10.1111/j.1365-2842.2006.01691.x

[10] Ueki K, et al. Evaluation of border movement of the mandible before and after orthognathic surgery using a kinesiograph. J Craniomaxillofac Surg. 2020;48(5):477–82. 32229178 10.1016/j.jcms.2020.02.021

[11] Kataoka T, et al. The influence of craniofacial morphology on mandibular border movements. Cranio. 2013;31(1):14–22. 23461258 10.1179/crn.2013.003

[12] Wen-Ching Ko E, et al. Longitudinal observation of mandibular motion pattern in patients with skeletal Class III malocclusion subsequent to orthognathic surgery. J Oral Maxillofac Surg. 2012;70(2):e158–68. 22260918 10.1016/j.joms.2011.10.002

[13] Ueki K, et al. Changes in border movement of the mandible in skeletal Class III before and after orthognathic surgery. Int J Oral Maxillofac Surg. 2014;43(2):213–6. 23953770 10.1016/j.ijom.2013.07.740

[14] Weffort SY, de Fantini SM. Condylar displacement between centric relation and maximum intercuspation in symptomatic and asymptomatic individuals. Angle Orthod. 2010;80(5):835–42. 20578853 10.2319/090909-510.1 PMC8939028

[15] Cordray FE. Articulated dental cast analysis of asymptomatic and symptomatic populations. Int J Oral Sci. 2016;8(2):126–32. 27357324 10.1038/ijos.2015.44 PMC4932769

[16] Scrivani SJ, Keith DA, Kaban LB. Temporomandibular disorders. N Engl J Med. 2008;359(25):2693–705. 19092154 10.1056/NEJMra0802472

[17] Giuntini V, et al. Glenoid fossa position in Class II malocclusion associated with mandibular retrusion. Angle Orthod. 2008;78(5):808–12. 18298205 10.2319/073007-353.1

[18] Paknahad M, Shahidi S, Abbaszade H. Correlation between condylar position and different sagittal skeletal facial types. J Orofac Orthop. 2016;77(5):350–6. 27357584 10.1007/s00056-016-0039-z

[19] Chae JM, et al. Evaluation of condyle-fossa relationships in adolescents with various skeletal patterns using cone-beam computed tomography. Angle Orthod. 2020;90(2):224–32. 31638857 10.2319/052919-369.1 PMC8051241

[20] Lobo F, et al. Imaginology Tridimensional Study of Temporomandibular Joint Osseous Components According to Sagittal Skeletal Relationship, Sex, and Age. J Craniofac Surg. 2019;30(5):1462–5. 31299744 10.1097/SCS.0000000000005467

[21] Vitral RW, et al. Computed tomography evaluation of temporomandibular joint alterations in patients with class II division 1 subdivision malocclusions: condyle-fossa relationship. Am J Orthod Dentofac Orthop. 2004;126(1):48–52. 10.1016/j.ajodo.2003.06.012 15224058

[22] Li J, et al. Dental, skeletal asymmetries and functional characteristics in Class II subdivision malocclusions. J Oral Rehabil. 2015;42(8):588–99. 25944587 10.1111/joor.12303

[23] Santander P, et al. Comprehensive 3D analysis of condylar morphology in adults with different skeletal patterns - a cross-sectional study. Head Face Med. 2020;16(1):33. 33256789 10.1186/s13005-020-00245-z PMC7708118

[24] Alhammadi MS, Fayed MS, Labib A. Three-dimensional assessment of temporomandibular joints in skeletal Class I, Class II, and Class III malocclusions: Cone beam computed tomography analysis. J World Federation Orthodontists. 2016;5(3):80–6.

[25] Hasebe A, et al. Comparison of condylar size among different anteroposterior and vertical skeletal patterns using cone-beam computed tomography. Angle Orthod. 2019;89(2):306–11. 30475648 10.2319/032518-229.1 PMC8120880

[26] Zhang Y, et al. Three-dimensional condylar positions and forms associated with different anteroposterior skeletal patterns and facial asymmetry in Chinese adolescents. Acta Odontol Scand. 2013;71(5):1174–80. 23294119 10.3109/00016357.2012.757359

[27] Ma Q, et al. Temporomandibular condylar morphology in diverse maxillary-mandibular skeletal patterns: A 3-dimensional cone-beam computed tomography study. J Am Dent Assoc. 2018;149(7):589–98. 29655707 10.1016/j.adaj.2018.02.016

[28] Mohsen AM, et al. Three-dimensional evaluation of the mandibular condyle in adults with various skeletal patterns. Korean J Orthod. 2023;53(2):67–76. 36806192 10.4041/kjod22.076 PMC10040293

[29] Khademi B, et al. Comparison of Glenoid Fossa Morphology Between Different Sagittal Skeletal Pattern Using Cone Beam Computed Tomography. J Craniofac Surg. 2020;31(8):e789–92. 33136913 10.1097/SCS.0000000000006750

[30] Fan Y, et al. 3D assessment of mandibular skeletal effects produced by the Herbst appliance. BMC Oral Health. 2020;20(1):117. 32299402 10.1186/s12903-020-01108-4 PMC7164294

[31] Koide D, et al. Morphological changes in the temporomandibular joint after orthodontic treatment for Angle Class II malocclusion. Cranio. 2018;36(1):35–43. 28198654 10.1080/08869634.2017.1285218

[32] Alhammadi MS, Fayed MS, Labib A. Three-dimensional assessment of condylar position and joint spaces after maxillary first premolar extraction in skeletal Class II malocclusion. Orthod Craniofac Res. 2017;20(2):71–8. 28150380 10.1111/ocr.12141

[33] Bayirli B, Vaden JL, Johnston LE Jr. Long-term mandibular skeletal and dental effects of standard edgewise treatment. Am J Orthod Dentofac Orthop. 2013;144(5):682–90. 10.1016/j.ajodo.2013.07.008 24182584

[34] Sójka A, et al. Evaluation of Mandibular Movement Functions Using Instrumental Ultrasound System. J Prosthodont. 2017;26(2):123–8. 26488230 10.1111/jopr.12389

[35] Zhang Y, Xu X, Liu Z. Comparison of Morphologic Parameters of Temporomandibular Joint for Asymptomatic Subjects Using the Two-Dimensional and Three-Dimensional Measuring Methods. J Healthc Eng. 2017;2017:p5680708. 10.1155/2017/5680708 PMC5434231 29065621

[36] Frahm J, Voit D, Uecker M. Real-Time Magnetic Resonance Imaging: Radial Gradient-Echo Sequences With Nonlinear Inverse Reconstruction. Invest Radiol. 2019;54(12):757–66. 31261294 10.1097/RLI.0000000000000584

[37] Mouchoux J, et al. Reliability of landmark identification for analysis of the temporomandibular joint in real-time MRI. Head Face Med. 2024;20(1):10. 38365709 10.1186/s13005-024-00411-7 PMC10874088

[38] Simmons HC 3rd, Oxford DE, Hill MD. The prevalence of skeletal Class II patients found in a consecutive population presenting for TMD treatment compared to the national average. J Tenn Dent Assoc. 2008;88(4):16–8. quiz 18 – 9. 19248341

